# Physicians’ Trust in the FDA’s Use of Product-Specific Pathways for Generic Drug Approval

**DOI:** 10.1371/journal.pone.0163339

**Published:** 2016-10-21

**Authors:** Aaron S. Kesselheim, Wesley Eddings, Tara Raj, Eric G. Campbell, Jessica M. Franklin, Kathryn M. Ross, Lisa A. Fulchino, Jerry Avorn, Joshua J. Gagne

**Affiliations:** 1 Program On Regulation, Therapeutics, And Law (PORTAL), Division of Pharmacoepidemiology and Pharmacoeconomics, Department of Medicine, Brigham and Women’s Hospital and Harvard Medical School, Boston, MA, United States of America; 2 Mongan Institute for Health Policy, Massachusetts General Hospital, Boston, MA, United States of America; 3 American Board of Internal Medicine, Philadelphia, PA, United States of America; Michigan State University, UNITED STATES

## Abstract

**Background:**

Generic drugs are cost-effective versions of brand-name drugs approved by the Food and Drug Administration (FDA) following proof of pharmaceutical equivalence and bioequivalence. Generic drugs are widely prescribed by physicians, although there is disagreement over the clinical comparability of generic drugs to brand-name drugs within the physician community. The objective of this survey was to assess physicians' perceptions of generic drugs and the generic drug approval process.

**Methods and Findings:**

A survey was administered to a national sample of primary care internists and specialists between August 2014 and January 2015. In total, 1,152 physicians comprising of internists with no reported specialty certification and those with specialty certification in hematology, infectious diseases, and endocrinology were surveyed. The survey assessed physicians’ perceptions of the FDA’s generic drug approval process, as well as their experiences prescribing six generic drugs approved between 2008 and 2012 using product-specific approval pathways and selected comparator drugs. Among 718 respondents (62% response rate), a majority were comfortable with the FDA’s process in ensuring the safety and effectiveness of generic drugs overall (91%) and with letting the FDA determine which tests were necessary to determine bioequivalence in a particular drug (92%). A minority (13–26%) still reported being uncomfortable prescribing generic drugs approved using product-specific pathways. Overall, few physicians heard reports of concerns about generic versions of the study drugs or their comparators, with no differences between the two groups. Physicians tended to hear about concerns about the safety or effectiveness of generic drugs from patients, pharmacists, and physician colleagues.

**Conclusions:**

Physicians hold largely positive views of the FDA's generic drug approval process even when some questioned the performance of certain generic drugs in comparison to brand-name drugs. Better education about the generic drug approval process and standards may alleviate concerns among the physician community and support the delivery of cost-effective health care.

## Introduction

Low-cost generic drugs generate major cost savings for the nation’s health care system, providing more than $931 billion in savings in the last decade.[1] At a time when US health care spending is on the rise, growing 3.6 percent and reaching $2.9 trillion in 2013 ($9,255 per person),[2] generic drugs remain a potential under-utilized source of cost saving. According to one national study examining generic drug substitution rates, generics could decrease overall US drug costs by 11%.[3,4]

The Food and Drug Administration (FDA) approves generic drugs on the basis of comparisons with the brand-name version that show pharmaceutical equivalence and bioequivalence. Generic products are considered pharmaceutical equivalents of the brand-name version if they contain the same active ingredient, are of the same dosage form, route of administration and are identical in strength or concentration. Bioequivalence is defined as the absence of significant differences in the availability of the active ingredient at the site of drug action.[5,6]

Bioequivalence studies usually compare pharmacokinetic measures in healthy volunteers, in fasting or fed states as appropriate, using a single dose of the drug, or a single exposure of the generic test and brand reference products. Numerous studies have confirmed that generic drugs certified as bioequivalent have similar clinical effects to the original brand name product.[7–9]

However, some physicians still question whether bioequivalence implies clinical equivalence. Controversy over the clinical comparability of generic drugs to brand name drugs is particularly high among "narrow therapeutic index" (NTI) drugs in which small differences in dose or blood concentration may lead to serious therapeutic failures or adverse drug reactions.[10–13] Other physicians have criticized the scope of the FDA's oversight, noting that the FDA does not have sufficient resources to confirm the quality of manufacturing of all generic drugs, particularly those manufactured outside the US.

Despite these concerns, payors and policymakers frequently urge physicians to prescribe generic drugs over brand-name drugs, when both options are available.[3,6,14–18]^,^ However, we know very little about physicians' perceptions of generic drugs and the generic drug approval process. Small prior studies have found mixed results with some citing positive attitudes towards generics,[19,20] and others claiming negative opinions.[4] We conducted a national survey of physicians to address these issues.

## Methods

### Study goals

We sought to investigate physician perceptions about the generic drug approval process and views specifically related to six generic drugs approved using product-specific pathways. Our approach was to develop a novel survey instrument and administer it to a national sample of primary care internists and specialists who would be expected to have experience with prescribing these drugs. The project was approved by the Institutional Review Board at Brigham and Women’s Hospital and FDA Research Involving Human Subjects Committee (RIHSC). Both oversight committees approved the process of obtaining consent, which was implied by participation after reading a description of the survey goals.

### Study drugs

In a 2013 grant announcement, the FDA Office of Generic Drugs published a list of six generic products approved between 2008 and 2012 using product-specific methods for assessing equivalence[21]: acarbose tablet (Precose, Bayer), vancomycin capsule (Vancocin, ViroPharma), sodium ferric gluconate injection (Ferrlecit, Sanofi-Aventis), enoxaparin injection (Lovenox, Sanofi-Aventis), calcitonin salmon nasal spray (Miacalcin, Novartis), and venlafaxine ER tablet (Effexor XR, Wyeth).

As some drugs have unique characteristics, product-specific approaches are needed to establish bioequivalence. There were four main rationales for the product-specific approval pathways used in these six cases. First, one drug (venlafaxine ER tablets) was known to be associated with severe nausea and vomiting in fasting patients, so the FDA only required in vivo testing under fed conditions.[22] Second, two drugs (acarbose tablet and vancomycin capsule) act locally in the intestine without being absorbed into the bloodstream,[23,24]so the FDA accepted *in vitro* studies alone to establish bioequivalence, as long as generic formulations were qualitatively and quantitatively the same as the brand-name reference drug.[25,26]Third, calcitonin salmon is a polypeptide, so its brand-name manufacturer argued a generic version should be subject to clinical trials prior to approval. But the FDA decided that clinical trials were not required, permitting approval based on more extensive than usual *in vitro* characterizations about the spray product or *in vivo* pharmacokinetic studies. Fourth, sodium ferric gluconate[27] and enoxaparin[28] are complex molecules made via unique production pathways, and their manufacturers argued that generic versions should duplicate the manufacturing process or conduct formal clinical trials. The FDA permitted generic approval based on extensive in vitro and in vivo testing without repeating clinical trials. All of these cases involved formal petitions[23,29–32] and lawsuits[33–37] by the affected manufacturers and publications describing the controversy in the medical literature and lay press.[38,39]

### Survey design

These questions were part of a larger survey about knowledge and perceptions of generic drugs and assessed physicians’ perceptions of the FDA’s generic drug approval process, as well as their experiences prescribing the 6 generic drugs of interest and selected comparator drugs. The questions were created based on an environmental literature scan with a survey development team including specialists in survey design, pharmacy law and policy, and pharmaceutical health services research and pre-tested, discussed, and adjusted based on a convenience sample of 4 physicians. See **S1 Appendix** for the survey questions.

#### Perceptions of generic drug approval and trust in FDA

We first asked physicians to judge how familiar they were with the FDA’s approval processes for brand-name and generic drugs (very familiar, familiar, a little familiar, not familiar at all). We then queried participants about their attitudes regarding FDA approval of generic drugs. We asked how comfortable they were “letting the FDA decide what tests are needed to prove brand-name and generic drugs are bioequivalent” and how comfortable they were that the FDA’s process ensured the safety and effectiveness of generic drugs, using a 4-point Likert scale ranging from “very comfortable” and “somewhat comfortable” to “somewhat uncomfortable” and “very uncomfortable.” On the same response scale, we next asked how comfortable they would feel if the FDA decided not to require a standard bioequivalence test that caused “significant side effects for participants” and how comfortable they would be prescribing a generic drug approved using a product-specific pathway that required “fewer tests.”

#### Hypothetical scenarios of generic drugs approved via product-specific regulatory pathways

Next, we presented physicians with 4 scenarios mirroring the four product-specific generic drug approval pathways of interest. The first described a hypothetical generic drug approved based on studies in fed patients but without testing on an empty stomach due to known side effects (modeled after venlafaxine extended release). Scenario 2 described a hypothetical generic drug that acted locally in the gastrointestinal tract, leading the FDA to require formulation sameness and *in vitro* dissolution similarity but forgo *in vivo* testing (acarbose and vancomycin). Scenario 3 described a more complex drug approved based on physicochemical characterization, composition sameness, and *in vivo* testing, but without requiring additional clinical trials (calcitonin salmon). Scenario 4 described a complex drug made via a unique manufacturing process also approved based on physicochemical characterization and in vivo testing but without requiring an identical manufacturing process or additional clinical trials (sodium ferric gluconate and enoxaparin).

After each scenario, we asked respondents whether they agreed or disagreed with the FDA’s decision to avoid certain tests and approve the drugs using product-specific bioequivalence pathways, and then asked on a 4-point Likert scale how comfortable respondents would be prescribing the hypothetical generic drug if it were approved by FDA (“very comfortable” to “very uncomfortable”).

#### Physicians’ experience with study and comparator generic drugs

In the next section, we assessed how often physicians could recall prescribing the 6 study drugs of interest in general in the last year (“never”, “1–10 different patients”, “11–20 different patients”, “>20 different patients”). We asked the same question of 6 additional drugs as comparators: four drugs with generics approved via non-product-specific bioequivalence pathways (bupropion [Wellbutrin], metronidazole oral tablets [Flagyl], repaglinide [Prandin], teriparatide [Forteo]) and two drugs with no generic versions available (iron sucrose injection [Venofer], dalteparin [Fragmin]). We selected each comparator to match as closely as possible the indications and mechanisms of action of one of the study drugs.

We then asked physicians whether they had heard reports of concerns about generic forms of the 12 drugs (yes/no). For each “yes” answer, we asked physicians to identify whether they heard the reports from the FDA, generic manufacturer, brand-name manufacturer, physician colleagues, patients, pharmacists, medical journals, Internet, or newspaper/radio/TV media. We then asked physicians the content of the concerns—whether the generic did not work as well, was not as safe, or was FDA-approved when it should not have been (yes/no for each). Finally, we asked whether the information changed physicians’ prescribing habits by leading them to stop prescribing the drugs, look for additional information on the issue, begin prescribing brand-name only, or did not change prescribing habits (yes/no for each).

#### Survey Sample

The American Board of Internal Medicine (ABIM) holds a list of diplomates with active certification and maintenance of certification. The dataset included demographic and medical training information and responses to the ABIM’s Practice Characteristics Survey.[40,41] We used these responses to supplement demographic information obtained from the survey.

As described previously, we randomly identified 1200 physicians who reported spending ≥40% of their time and ≥21 hrs/mo in patient care activities, and spending ≤50% of their time in the ICU, ED, or cardiac catheterization lab.[42] To obtain a range of primary care practitioners and specialists likely to prescribe the 6 drugs of interest, we identified 300 internists who reported no specialty certification, and 300 each who reported specialty certification in hematology, infectious diseases, and endocrinology. Selection of this sample size was based on a power calculation in which we estimated the true risk ratios describing the effect of a binary factor on comfort with generic drugs, and varied prevalences of that binary factor. Forty-eight potential participants had non-current contact information, leaving a sample size of 1152.

#### Survey Administration

Between August 2014 and January 2015, sample physicians received a postcard and three emails from ABIM indicating they had been randomly selected for a survey on generic drugs. Mailed hard copies of the survey were sent along with a cover letter describing the sponsoring institutions and lead investigators, a link to the on-line survey, an opportunity to opt out, and an offer of $50 upon completion. Non-responders received a mailed version along with a $5 bill and an offer for $45 upon completion. Remaining non-responders received a fourth email reminder. Physicians could respond via hard copy or electronically.

#### Statistical Analysis

We report estimated proportions and their confidence intervals, calculated using Wilson’s method. We used a chi-square test to compare the observed and expected counts. All analyses were done in Stata 14.1 (StataCorp LP, College Station TX).

## Results

Of the 1,152 physicians in the sample, 718 responded (124 hard copy and 594 online), for a response rate of 62%. **Table 1** shows the demographic and practice characteristics of the final analytic cohort.

**Table 1:** Characteristics of survey respondents.

Characteristic	Respondents, n/N*	Respondents, %
Total	718	100
*Sex*		
Male	374/687	54
Female	313/687	46
*Race/ethnicity*		
African American	32/675	5
Hispanic	40/675	6
Asian/Pacific Islander	179/675	27
Caucasian/White	393/675	58
Other	35/675	5
Primary practice area		
Internal medicine	182/643	28
Endocrinology	162/643	25
Infectious Diseases	174/643	27
Hematology	125/643	19
*Other*		
US medical school	387/639	61
Patient care >80% of professional time	376/643	58

* Denominators vary across questions because some respondents did not complete the entire survey.

### Physician knowledge and comfort with FDA drug approval process

About 88% of physicians reported being “familiar” or “a little familiar” with the brand-name drugs approval process (629/716), while only about 74% responded in the same way about the generic drug approval process (528/717). By contrast, 12% reported being “not familiar at all” with the brand-name approval process, and 26% reported being “not familiar at all” with the generic drug approval process (**Table 2**).

**Table 2:** Physicians’ familiarity and comfort with the FDA drug approval process.

	Respondents n/N* (%)
Familiarity with brand-name approval	
Very Familiar	62/716 (9)
Familiar	285/716 (40)
A little familiar	282/716 (39)
Not familiar at all	87/716 (12)
Familiarity with generic approval	
Very Familiar	24/717 (3)
Familiar	174/717 (24)
A little familiar	330/717 (46)
Not familiar at all	189/717 (26)
Level of comfort with the FDA approval process ensuring the safety and effectiveness of generic drugs	
Very comfortable	241/703 (34)
Somewhat comfortable	397/703 (56)
Somewhat uncomfortable	57/703 (8)
Very uncomfortable	8/703 (1)
Level of comfort with the FDA requiring fewer tests in approving generic drugs via product-specific pathways?	
Very comfortable	149/704 (21)
Somewhat comfortable	329/704 (47)
Somewhat uncomfortable	190/704 (27)
Very uncomfortable	36/704 (5)
Level of comfort with letting FDA determine tests needed for bioequivalence testing leading to generic drug approval	
Very comfortable	346/705 (49)
Somewhat comfortable	305/705 (43)
Somewhat uncomfortable	43 /705 (6)
Very uncomfortable	11/705 (2)
Level of comfort with approving generic without a specific bioequivalence test because of side effects it would cause	
Very comfortable	72/704 (10)
Somewhat comfortable	249/704 (35)
Somewhat uncomfortable	289/704 (41)
Very uncomfortable	94/704 (13)

* Denominators vary across questions because some respondents did not complete the entire survey.

With regard to generic drug approval and the use of product-specific pathways, an overwhelming majority of physicians were at least somewhat comfortable with the FDA’s process in ensuring the safety and effectiveness of generic drugs overall (638/703, 91%) and with letting the FDA determine which tests were necessary to determine bioequivalence in a particular drug (651/705, 92%). However, a somewhat smaller number of physicians (478/704, 68%) were comfortable with a hypothetical FDA decision to approve a generic drug based on fewer tests than normal, and an even smaller number were comfortable with the possibility that the FDA would forgo a specific bioequivalence test for a particular product because that test would cause side effects in research subjects (321/704, 46%).

### Hypothetical scenarios

When we tested physicians’ reactions to four different generic drug approval scenarios, we found that respondents generally favored the FDA performing additional tests if given the option (**Table 3**). In Scenario A, about half (53%) of respondents thought the FDA should have required fasting tests, even if the drug’s label stated that it should be taken with food and may cause safety concerns in healthy subjects during a bioequivalence fasting study, and in Scenario B, 65% reported that the FDA should have required extra blood level tests for a generic drug that works locally in the gastrointestinal tract without being absorbed into the bloodstream. Still, in Scenarios A and B, a majority of respondents reported that they would still be comfortable prescribing the FDA-approved drug without the additional tests (87% for A and 74% for B).

**Table 3:** Physicians’ perceptions of hypothetical scenarios of product-specific FDA approval processes for generic drugs.

	Respondents N/Total N*	95% Confidence Interval % (%, %)
Drug A: The FDA did not require fasting tests of the generic version before approval because drug may cause nausea and vomiting if taken on an empty stomach		
**FDA should have required extra fasting tests**		
Yes	365/695	53 (49, 56)
No	330/695	47 (44, 51)
**Comfortable prescribing FDA-approved version**		
Very/somewhat comfortable	607/697	87 (84, 89)
Very/somewhat uncomfortable	90/697	13 (11, 16)
Drug B: Drug works locally in the gastrointestinal tract, FDA approves generic version on similarity in physicochemical characteristics without requiring measurement of blood levels of drug		
**FDA should have required extra blood level tests**		
Yes	449/694	65 (61, 68)
No	245/694	35 (32, 39)
**Comfortable prescribing FDA-approved version**		
Very/somewhat comfortable	514/694	74 (71, 77)
Very/somewhat uncomfortable	180/694	26 (23, 29)
Drug C: Drug is complex large molecule, and FDA approves generic version with same composition and physicochemical properties from lab and human blood level tests		
**Reasonable conclusion for FDA scientists to draw**		
Yes	545/692	79 (76, 82)
No	147/692	21 (18, 24)
**Comfortable prescribing FDA-approved version**		
Very/somewhat comfortable	535/693	77 (74, 80)
Very/somewhat uncomfortable	158/693	23 (20, 26)
Drug D: Drug is complex large molecule made by a distinctive manufacturing process, and FDA approves generic version made using a similar (but not identical) process having same physical and chemical properties		
**Believe similar manufacturing process will produce an interchangeable drug**		
Yes	372/693	54 (50, 57)
No	321/693	46 (43, 50)
**Comfortable prescribing FDA-approved version**		
Very/somewhat comfortable	538/695	77 (74, 80)
Very/somewhat uncomfortable	157/695	23 (20, 26)

* Denominators vary across questions because some respondents did not complete the entire survey.

In Scenarios C and D, majorities of respondents did not see the need for additional testing on the complex drugs described (79% and 54%, respectively), in each case supporting the FDA’s decision to approve a generic based on *in vitro* and *in vivo* tests or and similarities in the physical and chemical properties. As in the first two scenarios, a majority of respondents reported being comfortable prescribing the hypothetical generic drugs after formal approval by the FDA (77% for Scenario C and 77% for Scenario D). Despite the confidence expressed by respondents in the FDA approval process, sizeable minorities of physicians reported not being comfortable prescribing the specific hypothetical generic drugs described (range: 13–26%).

### Reports of concerns about the 6 study drugs and comparators

Physicians in our sample varied in how often they prescribed the 6 study drugs and 6 comparators in the last year. Among the study drugs, many physicians had experience with venlafaxine extended release (43%), vancomycin capsules (51%), and enoxaparin (56%), while fewer had experience with sodium ferric gluconate (19%), acarbose (22%), and salmon calcitonin nasal spray (20%). Among the comparator drugs, the most frequently prescribed were repaglinide (63%) and metronidazole tablets (68%), while the least were dalteparin (13%) and iron sucrose injections (28%).

Overall, few physicians heard reports of concerns about generic versions of the study drugs. There were 26 physicians who reported hearing concerns about generic venlafaxine extended release (7%), 19 who reported hearing concerns about generic vancomycin capsules (5%), and 19 who reported hearing concerns about generic enoxaparin (4%). It was rare for physicians to report hearing concerns about generic versions of salmon calcitonin nasal spray (N = 13), acarbose (N = 8), and sodium ferric gluconate (N = 8). By contrast, somewhat larger numbers of physicians reported hearing concerns about generic versions of comparator drugs such as bupropion (N = 36), repaglinide (N = 60), teriparatide (N = 38) and metronidazole capsules (N = 14). A few physicians reported hearing concerns about generic versions of dalteparin (N = 4) and iron sucrose injection (N = 10) even though no generic versions of these products were available.

As seen in **Table 4**, physicians tended to hear concerns about the study generic drugs from patients, pharmacists, and physician colleagues. Such reports were least often attributed to FDA, the media (newspaper/radio/TV), and drug manufacturers (either brand-name or generic). For example, the most common source of concerns about venfalaxine extended release was patients (50% of reports), as compared to physician colleagues (23%), medical journals (23%), and pharmacists (19%). Patients were also a common source of reports of concerns about generic versions of the comparator drugs, along with physician colleagues. For example, 60 physicians reported hearing concerns about repaglinide, with 48 (80%) attributing the reports to patients, 13 (22%) to physician colleagues, and 10 (17%) to the brand-name manufacturer.

**Table 4:** Reports of concerns about study and comparator generic drugs and sources of concerns.

	Heard report of concern about generic	Reported prescribing in last year	Source of concerning report*								
			FDA	Generic mfr	Brand-name mfr	Physician colleagues	Pharmacist	Patient	Medical journal	Internet	Media
	N*	N	N (%)	N (%)	N (%)	N (%)	N (%)	N (%)	N (%)	N (%)	N (%)
***Study Drugs***											
Venlafaxine extended release	26	306	3 (12)	2 (8)	3 (12)	6 (23)	5 (19)	13 (50)	6 (23)	4 (15)	2 (8)
Salmon calcitonin nasal spray	13	144	3 (23)	0	3 (23)	3 (23)	2 (15)	1 (8)	3 (23)	3 (23)	1 (8)
Vancomycin capsules	19	368	4 (21)	1 (5)	1 (5)	9 (47)	9 (47)	6 (32)	2 (11)	2 (11)	3 (16)
Acarbose	8	157	2 (25)	2 (25)	1 (13)	2 (25)	2 (25)	2 (25)	3 (38)	0	0
Sodium ferric gluconate	8	137	1 (13)	1 (13)	1 (13)	2 (25)	4 (50)	1 (13)	3 (38)	4 (50)	0
Enoxaparin	19	400	3 (16)	0	4 (21)	6 (32)	6 (32)	5 (26)	5 (26)	4 (21)	4 (21)
Any study drug	78	630	22 (28)	12 (15)	15 (19)	32 (41)	25 (32)	29 (37)	26 (33)	20 (26)	12 (15)
***Comparator Drugs***											
Bupropion	36	334	3 (8)	3 (8)	3 (8)	12 (33)	8 (22)	22 (61)	1 (3)	8 (22)	4 (11)
Teriparatide	38	363	5 (13)	3 (8)	7 (18)	17 (45)	6 (16)	17 (45)	11 (29)	7 (18)	1 (3)
Metronidazole capsules	14	491	2 (14)	2 (14)	1 (7)	3 (21)	4 (29)	10 (71)	1 (7)	0	0
Repaglinide	60	452	3 (5)	2 (3)	10 (17)	13 (22)	7 (12)	48 (80)	7 (12)	7 (12)	4 (7)
Iron sucrose injection**	10	203	0	1 (10)	2 (20)	3 (30)	5 (50)	4 (40)	0	3 (30)	0
Dalteparin**	4	93	0	0	0	1 (25)	1 (25)	0	0	0	0
Any comparator	105	671	16 (15)	13 (12)	25 (24)	46 (44)	28 (27)	84 (80)	25 (24)	23 (22)	11 (10)

* Numbers add up to greater than the total number of physicians reporting hearing concerns about the generic versions of these drugs because answers were non-mutually exclusive. Other written-in responses included emergency medicine respondents, own experience as a patient

** Products had no generic comparators available in the US market at the time of the survey.

As **Table 5** shows, the nature of the reports of concerns was usually that the generic drug did not work as well as the brand-name version; by contrast, safety concerns and concerns about the FDA improperly approving the generic product were rarely mentioned. Physicians’ most common response to hearing these concerns was to seek additional information, but ultimately to not change their prescribing practice. In a minority of occasions, physicians stopped prescribing the drug or sought to ensure that only the brand-name version was dispensed.

**Table 5:** Reports of concerns about study and comparator generic drugs, nature of concern, and responses.

	Heard report of concern about generic	Nature of concern			Response			
		Did not work as well	Was not as safe	Should not have been approved	Stop prescribing	Seek more info	Prescribe brand-name only	No change
	N*	N (%)	N (%)	N (%)	N (%)	N (%)	N (%)	N (%)
***Study Drugs***								
Venlafaxine extended release	26	15 (58)	3 (12)	4 (15)	3 (12)	11 (42)	5 (19)	7 (27)
Salmon calcitonin nasal spray	13	7 (54)	1 (8)	1 (8)	3 (23)	4 (31)	0	5 (38)
Vancomycin capsules	19	9 (47)	2 (11)	2 (11)	1 (5)	6 (32)	3 (16)	7 (37)
Acarbose	8	2 (25)	0	1 (13)	0	4 (50)	0	4 (50)
Sodium ferric gluconate	8	1 (13)	5 (63)	1 (13)	0	4 (50)	0	2 (25)
Enoxaparin	19	5 (26)	4 (21)	6 (32)	0	7 (37)	1 (5)	10 (53)
***Comparator Drugs***								
Bupropion	36	27 (75)	5 (14)	4 (11)	1 (3)	21 (58)	5 (14)	16 (44)
Teriparatide	38	21 (55)	9 (24)	4 (11)	1 (3)	14 (37)	5 (13)	17 (45)
Metronidazole capsules	14	4 (29)	0	0	0	3 (21)	0	9 (64)
Repaglinide	60	29 (48)	7 (12)	2 (3)	0	22 (37)	5 (8)	29 (48)
Iron sucrose injection	10	1 (10)	5 (50)	1 (10)	1 (10)	3 (30)	0	4 (40)
Dalteparin	4	1 (25)	0	0	1 (25)	0	0	0

* Numbers may add up to greater than the total number of physicians reporting hearing concerns about the generic versions of these drugs because answers were non-mutually exclusive.

### Additional analyses

We paired each of the six study drugs with one or more of the primary practice areas from Table 1 most likely to be involved in prescribing the drug. Acarbose, for example, was paired with internal medicine and endocrinology. Table 6 shows that respondents in the associated practice areas were more likely to report concerns than physicians in practice areas who were less likely to prescribe the drug: for each drug the observed number of concerns in the third column exceeds the number expected by chance in the fourth column, and the difference is statistically significant (p = 0.002 by a chi-square test with six degrees of freedom).

**Table 6:** Specialists in the relevant primary practice areas for each drug reported more concerns.

Drug	Relevant specialties in survey*	Total N concerns^†^	N concerns from relevant specialties	Expected N concerns‡
Venlafaxine extended release	--	26	15	7.4
Salmon calcitonin nasal spray	Endocrinology	12	10	6.4
Vancomycin capsules	Infectious diseases	17	11	9.4
Acarbose	Endocrinology	6	5	3.2
Sodium ferric gluconate	Hematology	7	7	3.3
Enoxaparin	Hematology	14	13	6.7

* Physicians listed as primary care internal medicine practitioners were counted in each category.

^**†**^ Excluding physicians with unknown specialty areas

‡ Total number of concerns multiplied by proportion of respondents in the relevant specialties.

## Discussion

In this survey of physicians, we found that most claimed to possess at least some knowledge of the generic drug approval process. While a substantial minority of physicians reported hearing concerns about individual generic drugs, there was no association between the extent of such reports and any controversy relating to the FDA approval of the generic products at issue.

The hypothetical scenarios reflecting four product-specific drug approval pathways led to some seemingly paradoxical responses. While a majority of physicians preferred additional safety testing of generic drugs—even without any true physiological justification—many physicians also reported they would be comfortable prescribing the FDA-approved drug without additional tests. However, these results could also be further evidence of physicians’ confidence in the FDA setting the proper testing level for new generic drugs. Other surveys have found strong support among consumers for FDA decisionmaking,[43] and this survey extends those results to physicians as well.

These results suggest a major shift in physicians’ perceptions about generic drugs over the past decade. While a survey from 2009 found that about half of physicians were concerned about generic drugs,[4] we found wide confidence in the ‘FDA's review process for these products. One reason for this evolution could be the introduction of generic versions of large number of widely-used brand-name drugs during this period such as atorvastatin (Lipitor) and olanzapine (Zyprexa), which could help increase the confidence of physicians in the oversight of the generic drug market. In addition, the overall increase in generic utilization, resulting in part from payor strategies to influence generic utilization, could increase the familiarity and comfort with generic drug approval.

Despite the majority of physicians having a positive overall view of the FDA, a substantial minority (in the range of 13–26%) still reported being uncomfortable prescribing the generic drugs studied in this survey. Though we do not know the particular reason each individual physician had for preferring brand-name versions, such numbers represent a sizable proportion of lost opportunities for generic drug prescribing. It is well-known that generic drug use increases medication adherence and it has even been demonstrated to improve patients’ clinical outcomes.[44] Better educational outreach from the FDA to physicians about the clinical benefits that patients experience as a result of being prescribed generics may help overcome some of these negative perceptions.

However, outreach to physicians is unlikely to be sufficient. Our survey found that many physicians reported receiving negative reports about generic drugs, particularly from pharmacists and patients. Other sources of negative reports include the FDA, brand-name manufacturers, physician colleagues and the Internet. The FDA has not issued negative reports about the drugs of interest in this survey, so these results most likely relate to publicity around other situations in which generic manufacturers violated Good Manufacturing Practices.[45] The potential for such reports to have spillover effects in reinforcing negative attitudes about unrelated generics emphasizes the need for the FDA to continue to ensure the quality of the global generic drug production market, and to broadcast how it is doing that to prescribers. Greater resources will help the FDA succeed in this goal.

To the extent that brand-name drug marketing—particularly on the Internet—contributes to these perceptions as well, it is overseen by the FDA Office of Prescription Drug Promotion (OPDP), although its funding has long been insufficient to cover the vast prescription drug marketplace. If the source of improper negative reports about bioequivalent generic drugs can be tracked to a brand-name manufacturer, the OPDP should have authority to restrict the misleading promotion. One way OPDP learns about such activities is through the “Bad Ad” program in which it solicits examples of potentially false or misleading advertising from physicians or consumers.

The survey is limited in that the sample of physicians included mostly internists and three internal medicine specialties; importantly, it is possible that perceptions about generics differ based on physician specialty. Although some physicians reported hearing concerns about generics, we cannot confirm whether these reports or the physicians' response of seeking additional information without changing prescribing practice were legitimate. In particular, we did not examine prescribing frequencies of the six drugs of interest and the comparator drugs, which may account for differences in observed complaints about generic versions. However, other pharmacoepidemiologic studies of these drugs used large claims databases found no differences in switchbacks between these products and matched comparators.[46] Further research on the details regarding the sources of concern about generic drugs could provide insight into improving physicians’ views of generic drugs and confidence in the generic drug approval process.

Enabling the FDA to continue its generic drug approval process using product-specific bioequivalence tests, while adhering to scientific standards, is important to ensuring the delivery of cost-effective health care. Our survey showed that physicians hold largely positive views of the FDA’s decision-making during the generic drug approval process even when some patients and physician colleagues expressed concerns about the utility of generic drugs in comparison to brand-name drugs. Remaining concerns about generic drugs and the generic drug approval process among the physician community may be addressed through a combination of educating physicians better about the generic drug bioequivalence testing process, seeking out and correcting misinformation about generic drugs, and ensuring adequate regulatory pharmacovigilance of the generic marketplace to ensure early detections of any safety problems that do emerge.

## Supporting Information

S1 Appendix On-line Appendix. Full text of survey questions.
(DOCX)
