# Supplementary material for: Physicians’ Trust in the FDA’s Use of Product-Specific Pathways for Generic Drug Approval
Source: PLoS One. 2016 Oct 21;11(10):e0163339. doi: 10.1371/journal.pone.0163339 (PMC5074566; doi:10.1371/journal.pone.0163339)
Supplement: S1 Appendix — Full text of survey questions. (DOCX) [file pone.0163339.s001.docx]

**On-Line Appendix**

**Appendix Contents**

**Full text of survey questions**

**Full text of survey questions**

In this survey, when we refer to a **brand-name drug**, we mean a first-to-market product that is usually protected by one or more patents held by a single manufacturer.  A **generic drug** is an interchangeable version of that drug made by another manufacturer after the original patents expire.

How familiar are you with the Food and Drug Administration's (FDA’s) approval processes for **brand-name drugs**?

- Very familiar
- Familiar
- A little familiar
- Not familiar at all

How familiar are you with the Food and Drug Administration's (FDA’s) approval processes for **generic drugs**?

- Very familiar
- Familiar
- A little familiar
- Not familiar at all

As you know, the FDA has an established testing and approval process that both brand-name and generic drug products go through to ensure they are safe and effective for their intended uses before they can be prescribed to patients.   To obtain approval of its brand-name drug, a manufacturer conducts animal and human trials.   A generic product must have the same active ingredient, strength, dosage form, and method of administration as its corresponding brand-name version (though it may differ in shape, color, or size).  A generic manufacturer must demonstrate **bioequivalence**, meaning that the generic drug must reach the same levels in the body as the corresponding brand-name drug.  In establishing bioequivalence, the generic drug is commonly tested in animal and human subjects, but the FDA does not require a generic manufacturer to repeat clinical trials. The next set of questions will be about the bioequivalence testing process.

How comfortable are you letting the FDA decide what tests are needed to prove brand-name and generic drugs are **bioequivalent**?

- Very comfortable
- Somewhat comfortable
- Somewhat uncomfortable
- Very uncomfortable

If a human study that is routinely part of the **bioequivalence** testing process would cause significant side effects for participants in the case of a particular drug, the FDA may approve the generic version without requiring this study. How comfortable are you with this policy?

- Very comfortable
- Somewhat comfortable
- Somewhat uncomfortable
- Very uncomfortable

FDA conducts a medical and scientific assessment prior to each specific determination of **bioequivalence**, and also solicits public comments by manufacturers and patients. How comfortable are you with the FDA’s process in ensuring the safety and effectiveness of generic drugs?

- Very comfortable
- Somewhat comfortable
- Somewhat uncomfortable
- Very uncomfortable

Overall, if the FDA requires fewer tests for a particular generic drug, but still certifies it as **bioequivalent**, how comfortable would you be prescribing that drug?

- Very comfortable
- Somewhat comfortable
- Somewhat uncomfortable
- Very uncomfortable

Please consider these **hypothetical** scenarios and respond to the associated questions.

**Situation 1**: The label for **Drug A** says that it should not be taken on an empty stomach because it can result in nausea and vomiting.  Even though the FDA usually requires testing of new generic drugs on both a full and an empty stomach, in this case, the FDA did not require testing on an empty stomach before approving a generic version of **Drug A** because of the language on the label. Do you think the FDA should have required testing the drug on both a full and empty stomach, or was testing on only a full stomach appropriate?

- Should have required testing on full and empty stomach
- Testing only on a full stomach was appropriate

The FDA approves generic **Drug A** as bioequivalent, and requires the same label warning patients not to take the drug on an empty stomach. How comfortable are you prescribing generic **Drug A**?

- Very comfortable
- Somewhat comfortable
- Somewhat uncomfortable
- Very uncomfortable

**Situation 2**: Brand-name antibiotic **Drug B** is designed not to be absorbed into a patient’s bloodstream. **Drug B** works by remaining within the lumen of the digestive tract, which is the main site of its antibacterial action. With the special way this drug works, FDA approves the generic form of **Drug B** because it has the same composition and because the generic drug acts exactly the same way as the brand-name version in a simulated digestive tract. Because the drug is designed not to enter the systemic circulation, the FDA does not require measurement of drug blood levels of generic Drug B among the usual tests required for approval. Should the FDA have required the generic manufacturer of **Drug B** to measure levels of the drug in the blood though no meaningful level of drug is expected to be found?

- Yes, even though the drug is not absorbed
- No, I agree with the FDA's decision

The FDA does not require the manufacturer of generic **Drug B** to conduct new trials to examine clinical outcomes (as a reminder, such trials are never required of generic drug manufacturers). The FDA approves the generic as bioequivalent. How comfortable are you prescribing generic **Drug B**?

- Very comfortable
- Somewhat comfortable
- Somewhat uncomfortable
- Very uncomfortable

**Situation 3**: Brand-name **Drug C** is an intravenous drug that is shown to be effective and safe in clinical trials. **Drug C** is larger and has a more complex chemical structure than most drugs. A generic version of it has the same composition and physical and chemical properties as **Drug C**. After laboratory and human blood level tests for the generic form of **Drug C**, the FDA concludes it will work exactly the same in patients who need **Drug C**. Was this a reasonable conclusion for FDA scientists to draw?

- Yes
- No

In evaluating generic **Drug C**, the FDA does not require repeating the same clinical trials that were done for the brand-name version of Drug C. The FDA approves the generic as bioequivalent. How comfortable are you prescribing generic **Drug C**?

- Very comfortable
- Somewhat comfortable
- Somewhat uncomfortable
- Very uncomfortable

**Situation 4**: Brand-name **Drug D** is a complex large molecule made by a distinctive manufacturing process. A generic version of it was made using a similar (but not identical) process by a different manufacturer and was found to have the same physical and chemical properties as brand-name **Drug D**, and met FDA quality standards. Do you believe this similar manufacturing process will produce an interchangeable drug?

- Yes
- No

Regardless of the manufacturing process for the drug product, generic **Drug D** is shown to be bioequivalent with tests deemed necessary and adequate by the FDA (that is, it achieves the same blood levels). The FDA thus approved the generic. How comfortable are you prescribing generic **Drug D**?

- - Very comfortable
  - Somewhat comfortable
  - Somewhat uncomfortable
  - Very uncomfortable

Please answer the following questions for 10 drugs you **might** **have** **prescribed** in the last year. Questions 3a-3c apply **only** to those drugs that you have heard concerning reports about.

Approximately how often have you prescribed each of the drugs listed below **in the past year**? Please **circle your answer** for **each** drug.

| **Venlafaxine extended release (Effexor XR)** | Never | 1-10 different patients | 11-20 different patients | >20 different patients |
| --- | --- | --- | --- | --- |
| **Salmon Calcitonin nasal spray (Miacalcin, Fortical)** | Never | 1-10 different patients | 11-20 different patients | >20 different patients |
| **Vancomycin oral capsules (Vancocin)** | Never | 1-10 different patients | 11-20 different patients | >20 different patients |
| **Acarbose (Precose)** | Never | 1-10 different patients | 11-20 different patients | >20 different patients |
| **Sodium Ferric Gluconate injection (Ferrlecit, Nulecit)** | Never | 1-10 different patients | 11-20 different patients | >20 different patients |
| **Enoxaparin (Lovenox)** | Never | 1-10 different patients | 11-20 different patients | >20 different patients |
| **Bupropion (Wellbutrin)** | Never | 1-10 different patients | 11-20 different patients | >20 different patients |
| **Dalteparin (Fragmin)** | Never | 1-10 different patients | 11-20 different patients | >20 different patients |
| **Metronidazole oral tablets (Flagyl)** | Never | 1-10 different patients | 11-20 different patients | >20 different patients |
| **Repaglinide (Prandin)** | Never | 1-10 different patients | 11-20 different patients | >20 different patients |
| **Teriparatide (Forteo)** | Never | 1-10 different patients | 11-20 different patients | >20 different patients |
| **Iron Sucrose injection (Venofer)** | Never | 1-10 different patients | 11-20 different patients | >20 different patients |

Have you heard about any reports of concerns about the generic forms of any of these drugs? **Please circle yes or no**

| **Venlafaxine extended release (Effexor XR)** | Yes No |
| --- | --- |
| **Salmon Calcitonin nasal spray (Miacalcin,Fortical)** | Yes No |
| **Vancomycin oral capsules (Vancocin)** | Yes No |
| **Acarbose (Precose)** | Yes No |
| **Sodium Ferric Gluconate injection (Ferrlecit, Nulecit)** | Yes No |
| **Enoxaparin (Lovenox)** | Yes No |
| **Bupropion (Wellbutrin)** | Yes No |
| **Dalteparin (Fragmin)** | Yes No |
| **Metronidazole oral tablets (Flagyl)** | Yes No |
| **Repaglinide (Prandin)** | Yes No |
| **Teriparatide (Forteo)** | Yes No |
| **Iron Sucrose injection (Venofer)** | Yes No |

Please answer questions the three questions on the next 3 pages **for any drugs with the answer “yes” above**.  If you circled “no” for all of the drugs in question 2, please **skip the questions below**.

Where did you hear these reports of concerns about the generic forms of the drugs you selected? **Please** **write in the name of the relevant drug from the above list and then circle yes or no**

|  | Drug name: | Drug name: | Drug name: | Drug name: |
| --- | --- | --- | --- | --- |
| The FDA | Yes No | Yes No | Yes No | Yes No |
| The manufacturer making the **generic** drug | Yes No | Yes No | Yes No | Yes No |
| The manufacturer making the **brand-name** drug | Yes No | Yes No | Yes No | Yes No |
| Physician colleagues | Yes No | Yes No | Yes No | Yes No |
| Pharmacist | Yes No | Yes No | Yes No | Yes No |
| Patient | Yes No | Yes No | Yes No | Yes No |
| Medical journals | Yes No | Yes No | Yes No | Yes No |
| The Internet | Yes No | Yes No | Yes No | Yes No |
| Newspapers/radio/TV reports | Yes No | Yes No | Yes No | Yes No |

Other (write-in):

What did you hear? **Please** **write in the name of the relevant drug from the above list and then circle yes or no**

|  | Drug name: | Drug name: | Drug name: | Drug name: |
| --- | --- | --- | --- | --- |
| The generic drug was safe and effective | Yes No | Yes No | Yes No | Yes No |
| The generic drug did not work as well | Yes No | Yes No | Yes No | Yes No |
| The generic drug was not as safe | Yes No | Yes No | Yes No | Yes No |
| The generic drug was FDA approved as the same as the brand-name when it should not have been | Yes No | Yes No | Yes No | Yes No |

Other (write-in):

Did the information change your prescribing habits? **Please** **write in the name of the relevant drug from the above list and then circle yes or no**

|  | Drug name: | Drug name: | Drug name: | Drug name: |
| --- | --- | --- | --- | --- |
| I stopped prescribing the drug altogether | Yes No | Yes No | Yes No | Yes No |
| I looked for additional information about the issue | Yes No | Yes No | Yes No | Yes No |
| I began prescribing only the brand-name form | Yes No | Yes No | Yes No | Yes No |
| It did not change my medication prescribing | Yes No | Yes No | Yes No | Yes No |

Other (write-in):

**Section 6. Demographics**

1. My primary practice area is:

- Primary care
- Specialty practice

2. My clinical specialty is/are: [write in up to 3]

- ____________________
- ____________________
- ____________________

3. Please write the 5-digit U.S. ZIP code for the primary location in which you practice.

_______________________

4. Please describe your gender:

- Male
- Female

5. Please describe your race or ethnicity [select all those that apply]:

- African American
- Hispanic
- Asian/Pacific Islander
- Caucasian/White
- Other; write in: ____________________

6. In what year were you born?

_______________

7. In what year did you complete medical school?

_______________

8. In what year did you complete your residency?

_______________
